# Prognostic value of the expression of epithelial cell adhesion molecules in head and neck squamous cell carcinoma treated by definitive radiotherapy

**DOI:** 10.1093/jrr/rrz053

**Published:** 2019-07-30

**Authors:** Naoya Murakami, Taisuke Mori, Satoshi Nakamura, Seiichi Yoshimoto, Yoshitaka Honma, Takao Ueno, Kenya Kobayashi, Tairo Kashihara, Kana Takahashi, Koji Inaba, Kae Okuma, Hiroshi Igaki, Yuko Nakayama, Jun Itami

**Affiliations:** 1 Department of Radiation Oncology, National Cancer Center Hospital, 5-1-1 Tsukiji, Chuo-ku, Tokyo, Japan; 2 Department of Pathology and Clinical Laboratories, National Cancer Center Hospital, 5-1-1 Tsukiji, Chuo-ku, Tokyo, Japan; 3 Department of Head and Neck Oncology, National Cancer Center Hospital, 5-1-1, Tsukiji Chuo-ku, Tokyo, Japan; 4 Head and Neck Medical Oncology Division, National Cancer Center Hospital, 5-1-1 Tsukiji, Chuo-ku, Tokyo, Japan; 5 Department of Oral Health and Diagnostic Sciences, National Cancer Center Hospital, 5-1-1, Tsukiji Chuo-ku, Tokyo, Japan

**Keywords:** head and neck squamous cell carcinoma, EpCAM, primary radiation therapy, biomarker, BerEP4

## Abstract

A reliable biomarker can contribute to appropriate treatment selection in the management of head and neck squamous cell carcinoma (HNSCC). Recently, epithelial cell adhesion molecule (EpCAM) was shown to have prognostic features in several malignancies. However, it remains to be elucidated whether EpCAM predicts prognosis of HNSCC after radiotherapy. Therefore, the prognostic potential of EpCAM in HNSCC patients treated by radiotherapy was investigated in this study. All HNSCCs patients examined between January 2013 and December 2015 were analyzed for the expression of EpCAM. One hundred HNSCC patients were identified who were treated by primary radiotherapy. Intense expression of EpCAM was found in 29 HNSCC patients. Two-year overall survival (OS) for patients with intense EpCAM expression was 62.2%, whereas it was 87.9% for those without (*P* = 0.011). In multivariate analysis, intense EpCAM expression was found to be an independent prognostic factors for OS (*P* = 0.036). Overall, EpCAM was found to be an independent prognostic factor for HNSCC.

## INTRODUCTION

Surgery, radiation therapy, and chemotherapy are major treatment modalities in the management of squamous cell carcinoma of the head and neck (HNSCC), and primary radiation therapy with or without chemotherapy is a well-recognized curative treatment modality [1–3]; however, tumor resistance or recurrence after radiation therapy is occasionally experienced in daily practice, even in early-stage disease. Therefore, the biomarkers that can reliably predict prognosis of HNSCC patients treated by radiation therapy is of value and the study of such biomarkers has been undertaken for several decades. Epidermal growth factor receptor (EGFR), p53 (one of the most well-known tumor suppressor proteins), and p16 (a surrogate biomarker for human papillomavirus [HPV] infection) are established prognostic factors with regard to radiation therapy for HNSCCs [4–10]. Notably, it has been clearly shown that HPV-positive HNSCCs are highly radiosensitive; however, HPV-positive tumors are mostly found in the oropharynx [7, 10]. Therefore, another trustworthy prognostic factor that is valid for all anatomical sites of HNSCCs is needed.

Epithelial cell adhesion molecule (EpCAM) is a type I trans-membrane protein mediating Ca^2+^-independent homotypic cell–cell adhesion on the surface of the epithelia [11]. EpCAM is frequently expressed in a variety of normal human epithelial tissues, mostly on basolateral membrane, progenitor and stem cells, and carcinomas [12]. In malignant tumors, EpCAM is stably expressed or even up-regulated and contributes to malignant progression of disease [13, 14]. However, EpCAM is not expressed on malignancies of non-epithelial origin, but is expressed on human epithelial malignant tumors, predominantly on adenocarcinomas. Because expression of EpCAM in squamous cell carcinoma is not frequent, there are few reports that dealt with expression of EpCAM in squamous cell carcinomas [15–17]. Recently, the prognostic value of EpCAM on cancer treatment has been reported in several types of tumors [17–19], however, the prognostic value of EpCAM after radiation therapy is unknown.

Since January 2013, our group has evaluated p53, p16, and EpCAM expression on all consecutive HNSCCs tissue specimens. In our previous study, we reported that EpCAM was a response predictive biomarker for primary radiation therapy in early-stage glottic cancer patients [15]. In this study, we investigated whether EpCAM would be a prognostic factor for primary radiation therapy in HNSCC patients.

## MATERIALS AND METHODS

With the approval of our Institutional Review Board, we collected formalin-fixed, paraffin-embedded specimens from all of the HNSCC patients prior to treatment. The study period was from January 2013 to December 2015. All specimens were reviewed in our institution, and the histologic tumor types were classified according to the WHO criteria [20]. Among them, patients who received definitive radiation therapy to the primary site were extracted and further investigated. In this study, it was defined that primary tumor was not surgically removed, but was irradiated with definitive radiation.

### Immunohistochemical analysis

**Fig. 1.**
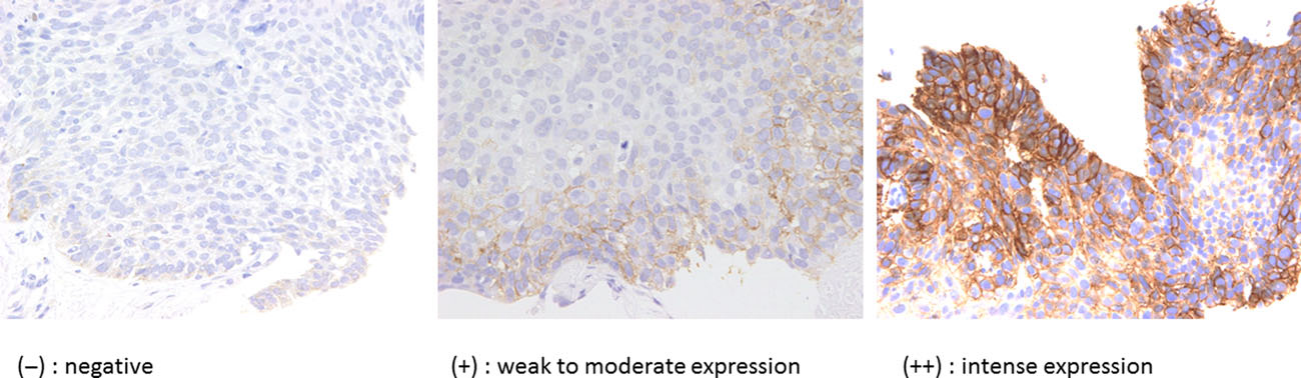
Typical staining patterns of BerEP4 are shown. The positivity of BerEP4 was defined as follows: (−) negative, (+) weak to moderate expression, and (++) intense expression.

All the judgement of immunohistochemical analysis was performed by pathologist T.M., one of co-authors of this article. Among several antibodies against EpCAM, BerEP4, which is frequently used in the diagnosis of malignant mesothelioma [21], was used to assess the expression of EpCAM in this study. Expression of BerEP4, p53, and p16 was assessed based on a biopsy specimen obtained before primary radiation therapy. The specific procedure of immunohistochemical analysis was described in our previous report [15]. Sections (4-μm thick) from a representative block of each tumor were deparaffinized. The sections were subjected to hematoxylin–eosin (H & E) and subsequent immunohistochemical staining using each of the primary antibodies: EpCAM (1:200, ab7504, Ber-EP4; Abcam, Cambridge, MA) [22]; p53 (1:400, DO-7; Dako, Carpinteria, CA); and p16 (1:50, p16ink4a, G175–405; BD Biosciences, San Jose, CA). Each section was exposed to 0.3% hydrogen peroxide for 15 min to block endogenous peroxidase activity. According to the protocol of the vendor, we used an automated stainer (Dako, Carpinteria, CA) ChemMate EnVision (Dako, Carpinteria, CA) method for staining biopsy samples. Appropriate positive and negative controls were used for each antibody. The immunohistochemistry of BerEP4 was evaluated by the percentage and the intensity of cell staining. The percentage of immune-positive stained cells was divided into three grades as follows: <10 % (−); 10–69% (+); and >70% (++). A typical staining pattern for BerEP4 is shown in Fig. 1. Additionally, expression of p53 and p16, well-recognized prognostic factors for HNSCCs, was also investigated. Intense expression of p53 in nucleus (accumulation of p53 protein in the tumor cells) or no expression (no production of p53 protein due to TP53 gene alteration) was defined to represent alteration of TP53 gene [23, 24]; otherwise, tumors were recognized to have no p53 mutation. For p16, only tumors having expression both in the cytoplasm and the nucleus were deemed to exhibit a HPV-infected pattern. According to our past experience, the immunohistochemical sensitivity and specificity of p16 were 94% and 82%, respectively, which was similar to a previous report [25].

### Treatment

Tumor T stage was evaluated according to the 7th edition of the American Joint Committee on Cancer/International Union against Cancer (AJCC/UICC) [26]. In T1–2N0 disease, in principle, no chemotherapy was used [15, 27]. From June 2009 neoadjuvant chemotherapy (NAC) was started as chemo-selection for patients with locally advanced HNSCC who otherwise required total laryngectomy or who expected severe postoperative pharyngeal dysfunction. If a favorable response was achieved after two to three cycles of induction chemotherapy, subsequently concurrent chemoradiation (cCRT) was followed with or without neck dissection. NAC was also applied to patients with far-advanced disease for whom it was impossible to separate metastatic lymph nodes from carotid artery or patients with N2c and/or lower neck metastasis whose possibility of developing distant metastasis soon after surgery was estimated to be very high. Agents used for NAC was either a combination of cisplatin (CDDP) and 5-fluorouracil (5-FU) or CDDP, 5FU, and docetaxel. Since April 2011 cCRT with tri-weekly CDDP 80 mg/m^2^, and since March 2013 Cetuximab-radiation according to Bonner protocol [28] was introduced in our institution for patients with advanced stage HNSCC. Because there exists no evidence supporting the superiority of Cetuximab-radiation over CDDP-based cCRT in advanced HNSCC, our first choice was CDDP-based cCRT and Cetuximab-radiation for patients with kidney dysfunction but still having a favorable performance status [29].

Radiotherapy of 2 Gy per fraction, five fractions per week, with 4- or 6-MV photons in either three-dimensional conformal radiotherapy (3DCRT) or intensity-modulated radiotherapy (IMRT) was prescribed. When target volume did not include a large volume of major salivary gland, oral cavity, larynx, pharynx, or optic apparatus, 3DCRT was selected; otherwise IMRT was applied [29]. For example, early-stage glottic cancer patients were treated by 3DCRT. Our IMRT procedure for HNSCC patients is described in a previous report [29]. For IMRT, we generally use a simultaneously integrated boost intensity-modulated radiotherapy (SIB-IMRT) with volumetric modulated arc therapy (VMAT) using a dynamic multileaf collimator (MLC) system (Varian Medical Systems, Palo Alto, CA). Primary tumor and lymph node metastasis received 70 Gy while 54 Gy was given to the prophylactic subclinical lymph node area. For nasopharyngeal carcinoma, 2-step IMRT was performed in which 46 Gy was delivered including gross tumor volume and prophylactic subclinical lymph node area followed by boost irradiation up to 70 Gy to the primary tumor and lymph node metastasis.

### Statistics

Overall survival rate (OS), progression-free survival rate (PFS), and in-field control rate (IFC) were calculated from the start of radiation therapy until the last follow-up visit or death from any cause, any disease recurrence, and histological or apparent radiological evidence of disease recurrence within the radiation field, respectively. Tumor recurrences found in the head and neck region covered by clinical target volume (CTV) 70 Gy/54 Gy/46 Gy were defined as in-field recurrence. IFCs calculated from the survival curves were estimated by using the Kaplan-Meier method and the differences were assessed by the log-rank test. A *P* value < 0.05 was considered statistically significant. Factors with *P* value < 0.05 were further analyzed in the multivariate analysis by Cox regression analysis. Cox proportional-hazards models were used to estimate hazard ratios. All statistical analyses were performed using SPSS Statistics (version 18.0; SPSS, Inc., Chicago, IL). This retrospective study was also approved by the Institutional Review Board of our hospital (approval number 2014-043) according to the ethical standards laid down in the Declaration of Helsinki. This study is dealing with a biomarker and adheres to REMARK criteria as listed in their guidelines [30].

## RESULTS

**Fig. 2.**
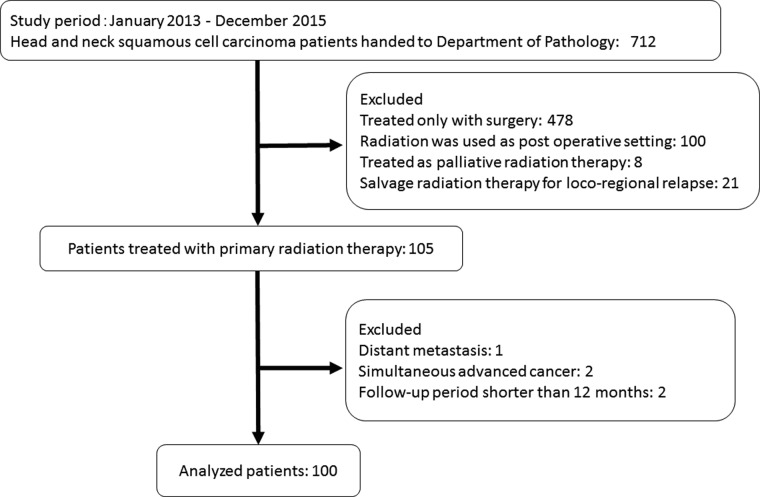
Consolidated Standards of Reporting Trials (CONSORT) flow diagram of consecutive HNSCC patients whose biopsy specimens were handed to our Department of Pathology from January 2013 to December 2015.

A Consolidated Standards of Reporting Trials (CONSORT) flow diagram is shown in Fig. 2. During the study period, 712 specimens were handed to the Department of Pathology. The following patients were excluded from the study: 478 patients treated with only surgery, 100 patients with postoperative radiotherapy, 8 patients with palliative radiotherapy, and 21 patients with salvage radiotherapy for loco-regional recurrence after initial therapy. A total of 105 specimens were identified and patients with distant metastasis, simultaneous advanced cancer, or follow-up period shorter than 12 months were further excluded. Finally, 100 HNSCC patients treated with primary radiation therapy were enrolled in this study.

Table 1 shows the demographic information of all the patients included in the study stratified by the expression of BerEP4. There were 12 HNSCCs from the oral cavity, 26 from the oropharynx, 19 from the hypopharynx, 28 from the larynx, 7 from nasopharynx, and 8 from other miscellaneous sites. Mutated pattern of p53 was observed in about 70% of patients. Among 26 oropharyngeal cancer patients, 18 (69.2%) patients were positive for p16 and among 27 HNSCC patients positive for p16, 18 (66.7%) patients had oropharyngeal cancer. Intense expression of BerEP4 was observed in about 30% of patients. It was found that tumors that had intensive expression of BerEP4 were more likely to have advanced disease. In terms of anatomic sites, distribution between intensive and non-intensive BerEP4 tumors were statistically different. Table 2 shows the demographic of oropharyngeal cancer patients stratified by p16 staining status in immunohistochemical analysis. Stratification by p16 was performed because it was well-known that oropharyngeal cancers associated with HPV infection are different from oropharyngeal cancers not related to HPV infection. Although there was no statistical difference, while p16 positive tumors rarely expressed intense BerEP4 expression, half of p16 negative tumors intensively expressed BerEP4. It was found that p16 positive tumors were more likely to have un-mutated p53 gene (*P* = 0.001).

**Table 1.** Patient and tumor characteristics

	BerEP4		*P*
	Intense expression (*n* = 29)	Non-intense expression (*n* = 71)	
Sex			
Male	27	61	0.261
Female	2	10	
Age, years			
Median, range	67 (18–79)	67 (32–86)	0.472
Primary site			
Oral cavity	1	11	<0.001^*^
Oropharynx	7	19	
Hypopharynx	7	12	
Larynx	6	22	
Nasopharynx	5	2	
Others	3	5	
T-classification			
T1	4	16	<0.001^*^
T2	11	30	
T3	10	17	
T4	4	7	
X	0	1	
N-classification			
N0	12	47	<0.001^*^
N1	2	3	
N2	14	18	
N3	1	3	
Stage			
I	2	12	<0.001^*^
II	7	19	
III	5	15	
IV	15	25	
p53			
Un-mutated	7	N4	0.343
Mutated	22	47	
p16			
HPV uninfected pattern	22	51	0.68
HPV infected pattern	7	20	

HPV = human papillomavirus.

**Table 2.** Demographic of oropharyngeal cancer patients (*n* = 26) divided by p16 status

	p16		*P*
	HPV infected pattern (*n* = 18)	HPV uninfected pattern (*n* = 8)	
Sex			
Male	13	8	0.130
Female	5	0	
Age, years			
Median, range	64 (53–83)	72 (58–78)	0.353
T-classification			
T1	3	1	0.534
T2	7	3	
T3	7	2	
T4	1	2	
N-classification			
N0	1	3	0.100
N1	0	0	
N2	16	5	
N3	1	0	
Stage			
I	0	0	0.094
II	1	2	
III	0	1	
IV	17	5	
p53			
Un-mutated	15	0	0.001^*^
Mutated	3	7	
BerEP4			
Intense expression	3	4	0.101
Non-intense expression	15	4	

HPV = human papillomavirus

Table 3 shows the treatment details. Four patients had large or multiple neck lymph node enlargement and they underwent preceding neck dissection prior to definitive radiation therapy. About half of patients received concurrent systemic therapy. Eleven patients with tongue cancer were treated with either high-dose rate or low-dose rate interstitial brachytherapy. Nineteen out of 23 patients treated by 3DCRT were glottic cancer patients. All the patients treated with external beam radiation therapy received >54 Gy. The majority of patients were treated with IMRT (66%).

**Table 3.** Treatment details

	*n*
Preceding ND	
Yes	4
No	96
NAC	
Yes	14
No	86
Concurrent systemic therapy	
Yes	54
No	46
RT technique	
3DCRT	23
IMRT	66
Brachytherapy	11
Total radiation dose (Gy)	
Median, range	70 (54–80)

ND = neck dissection, NAC = neoadjuvant chemotherapy, RT = radiation therapy, 3DCRT = three dimensional radiation therapy, IMRT = intensity modulated radiation therapy.

The median follow-up period for patients still alive at last contact was 22.4 months (range, 12.4–38.9). Two-year OS, PFS, and IFC were 80.7%, 60.6%, and 75.0%, respectively.

A summary of univariate and multivariate analysis for IFS, PFS, and OS is shown in Table 4. In univariate analysis, the IFC of patients treated a by non-IMRT technique was significantly superior to that of patients treated by an IMRT technique (2-year IFC 88.2% vs 68.4%, respectively, *P* = 0.05). It was estimated that the majority of patients treated by non-IMRT were patients with early-stage glottic cancer, local control of which was excellent. Likewise, patients treated by concurrent systemic therapy showed a trend toward inferior IFC compared with patients treated with radiotherapy alone (2-year IFC 67.7% vs 84%, respectively, *P* = 0.055). This result, again, was supposed to be influenced by confounding bias that more advanced stage tumors were treated with concurrent systemic therapy. Subgroup analysis was performed only for advanced patients with Stage III and IV with regards to the application of systemic therapy, however, the usage of systemic therapy did not positively correlate with their clinical outcome, presumably because of the limited number of advanced patients included in the subgroup analysis.

**Table 4.** Hazard radios for in-field control (IFC), progression-free survival (PFS), and overall (OS) rates

Covariate				*P* in univariate analysis	*P* in multivariate analysis	Hazard ratio (95% CI)
**In-field control rate**		**2-year IFC (%)**				
Age	<67 vs ≥67	78.6	71.7	0.339		
Sex	Male vs female	72.6	91.7	0.165		
T-category	T1/2 vs 3/4	84.6	59.2	0.004^*^	0.167	0.482 (0.171–1.356)
N-category	0/1 vs 2/3	77.1	71.4	0.602		
Stage	1/2 vs 3/4	86.7	67.4	0.020^*^	0.524	0.632 (0.154–2.594)
NAC	Yes vs no	59.5	77.2	0.258		
RT technique	IMRT vs non-IMRT	68.4	88.2	0.050^*^	0.663	0.622 (0.073–5.281)
Systemic therapy	Yes vs no	67.7	84.0	0.055		
	Stage III/IV yes (*n* = 32) vs no (*n* = 6)	65.7	77.8	0.633		
RT total dose	<70 Gy vs ≥70 Gy	95.0	70.1	0.033^*^		
p53	Mutated vs un-mutated	72.6	79.2	0.357		
p16	HPV infected vs un-infected pattern	76.7	74.0	0.360		
BerEP4	Intense vs non-intense expression	52.2	84.0	0.001^*^	0.009^*^	3.069 (1.328–7.092)
**Progression-free survival rate**		**2-year PFS (%)**				
Age	<67 vs ≥67	59.8	61.6	0.983		
Sex	Male vs female	58.2	75.0	0.316		
T-category	T1/2 vs 3/4	66.4	50.4	0.134		
N-category	0/1 vs 2/3	67.2	49.9	0.136		
Stage	1/2 vs 3/4	71.0	54.2	0.082	0.111	0.566 (0.281–1.139)
NAC	Yes vs no	42.9	63.0	0.317		
RT technique	IMRT vs non-IMRT	54.9	72.4	0.168		
Systemic therapy	Yes vs no	58.6	63.7	0.659		
	Stage III/IV yes (*n* = 32) vs no (*n* = 6)	50.8	50.0	0.629		
RT total dose	<70 Gy vs ≥70 Gy	77.4	55.6	0.100		
p53	Mutated vs un-mutated	57.3	66.5	0.297		
p16	HPV infected vs un-infected pattern	58.3	60.7	0.789		
BerEP4	Intense vs non-intene expression	42.6	67.8	0.025^*^	0.037^*^	1.976 (1.040–3.755)
**Overall survival rate**		**2-year OS (%)**				
Age	<67 vs ≥67	80.0	81.9	0.968		
Sex	Male vs female	80.4	83.3	0.725		
T-category	T1/2 vs 3/4	90.1	65.6	0.021^*^	0.547	0.850 (0.502–1.441)
N-category	0/1 vs 2/3	86.3	71.2	0.061		
Stage	1/2 vs 3/4	95.0	71.7	0.017^*^	0.141	0.341 (0.081–1.428)
NAC	Yes vs no	68.8	82.7	0.356		
RT technique	IMRT vs non-IMRT	76.5	89.2	0.188		
Systemic therapy	Yes vs no	74.4	88.9	0.176		
	Stage III/IV yes (*n* = 32) vs no (*n* = 6)	72.8	66.7	0.546		
RT total dose	<70 Gy vs ≥70 Gy	94.7	77.3	0.078		
p53	Mutated vs un-mutated	76.4	90.2	0.081		
p16	HPV infected vs un-infected pattern	79.7	81.5	0.695		
BerEP4	Intense vs non-intense expression	62.2	87.9	0.011^*^	0.036^*^	2.597 (1.064–6.335)

NAC = neoadjuvant chemotherapy, RT = radiation therapy, IMRT = intensity modulated radiation therapy, HPV = human papillomavirus.The *p* values which are statistically significant are marked with asterisks.

Although p16 positivity was not a predictive factor for IFC, PFS, and OS, when the analysis was limited to 26 patients with oropharyngeal cancer, it was found that there was a trend toward better PFS in patients positive for p16 compared with patients negative for p16 (2-year PFS 66.7% vs 42.9%, respectively, *P* = 0.084).

There was a trend toward inferior OS for patients with mutated p53 compared with OS for patients with un-mutated p53 (2-year OS 76.4% vs 90.2%, respectively, *P* = 0.081).

In the multivariate analysis, only intensive expression of BerEP4 was a significant independent determinant of OS, PFS, and IFC (Table 4).

## DISCUSSION

Since January 2013, all consecutive HNSCC patients whose pathologic specimens were handed to our Department of Pathology were analyzed for the expression of EpCAM, p16, and p53. It was demonstrated through the current study that, besides T-category and Stage, intensive expression of EpCAM was an independent prognostic factor for primary radiation therapy for patients with HNSCC.

p16, one of thesurrogate markers of HPV infection, is a well-known favorable prognostic biomarker in oropharyngeal cancer [10]. Although, favorability of p16 was not demonstrated in this study, a trend for better PFS for patients with HPV-infected pattern was shown when the analysis was limited to 26 oropharyngeal cancer patients. Therefore, it was supposed that the reason for this result was because only a limited number of oropharyngeal cancer patients were entered into this study. As shown in table 2, interaction between p16, p53, and EpCAM was analyzed in oropharyngeal cancer patients. Although it did not reach statistical significance, while only a few patients intensively expressed EpCAM in p16 positive tumors, half of p16 negative tumors intensively expressed EpCAM and it was found that p16 negative tumors were more likely to have p53 mutation, suggesting different carcinogenesis scenarios between p16 positive and negative tumors.

In our previous report, we could only show that EpCAM was an effect predictor of primary radiation therapy for patients with early-stage glottic cancer through a case-control study [15], and we could not show that EpCAM was a prognostic factor, that is related to OS, for primary radiation therapy because the follow-up period was short since the study was performed shortly after the start of our project. In the current study, we demonstrated that EpCAM was not only an effect predictor but also a prognostic factor for primary radiation therapy in patients with HNSCC.

It is well-known that adenocarcinoma is more radio-resistant than squamous cell carcinoma at other anatomical sites [31, 32] and EpCAM is essentially expressed in all adenocarcinomas [22]. Therefore, it is understandable that HNSCC, which has characteristics of adenocarcinoma, is more radio-resistant. EpCAM is a cancer stem cell marker [12], which has the potential for self-renewal, pluripotency, and is resistant to chemotherapy or radiation therapy. Therefore, it is also understandable that HNSCC with intense expression of EpCAM showed radio-resistance.

BerEP4, one of the monoclonal antibodies recognizing EpCAM, has long been used for the diagnosis of mesotheliomas and, therefore, is not very expensive and is available in most hospitals [21]. It is meaningful that it is possible to predict the prognosis of HNSCC patients who will undergo primary radiation therapy using such an easy-to-access and inexpensive agent.

Recently precision medicine has begun to be performed in the management of several types of cancers such as breast cancer [33], lung cancer [34], and melanoma [35]. In oropharyngeal cancer, it was shown that prognosis of HPV-related tumors were generally favorable [10]. However, other than oropharyngeal cancer, no clinically relevant biomarker is used in daily clinical practice. It was shown in the current study that EpCAM could be a potential candidate as a biomarker in the management of HNSCC. Since it was demonstrated that HNSCC tumors that express intensive EpCAM have worse prognosis after primary radiation therapy, it will be interesting to compare clinical outcome of primary surgery followed by postoperative radiation therapy to that of primary radiation therapy for this population of patients in future studies. If it is possible to show that primary surgery followed by postoperative radiotherapy is more effective than primary radiation therapy for HNSCC expressing EpCAM intensively, a more individualized treatment approach could be offered to patients according to their specific tumor characteristics.

There are several limitations to this study. Although it is recommended that molecular analysis should be performed, because immune-histochemical analysis cannot distinguish the several different types of mutations [36], the gene itself was not analyzed in this study. Therefore, the result of this study should be interpreted with caution. However, it could still be profitable to use immune-histochemical analysis because of its easiness and availability. Because only limited number of patients were included in this analysis, all sites were analyzed at the same time. Since the clinical behaviors of specific anatomical sites differ from each other, such analysis should be performed at each specific anatomical site. As shown in table 1, intensive expression of EpCAM was associated with more advanced disease which requires administration of systemic chemotherapy. However, in the multivariate analysis it was found that EpCAM was the only independent factor associated with IFC, PFS, and OS (table 4). The follow-up period of this study was relatively short, therefore, further research is required. And finally, this study was a retrospective study from a single institution. To validate the result of this study a future prospective clinical trial is needed, because it is well known that numerous initially promising biomarker studies resulted in negative consequences in phase III prospective clinical trials [30, 37]. If the negative prognostic impact of EpCAM and p53 is validated, a treatment strategy should be developed not only based on tumor site and Stage, but also on EpCAM and p53 status.

## CONCLUSION

The intense expression of EpCAM was found to be an independent adverse prognostic factor for patients with HNSCC treated by primary radiation therapy.

## CONFLICT OF INTEREST

The authors declare that they have no conflict of interest.

## FUNDING

This study was partially supported by the Japan Agency for Medical Research and Development, AMED, the National Cancer Center Research and Development Fund (26-A-18 and 26-A-28).
